# Causal Inference of Genetic Variants and Genes in Amyotrophic Lateral Sclerosis

**DOI:** 10.3389/fgene.2022.917142

**Published:** 2022-06-22

**Authors:** Siyu Pan, Xinxuan Liu, Tianzi Liu, Zhongming Zhao, Yulin Dai, Yin-Ying Wang, Peilin Jia, Fan Liu

**Affiliations:** ^1^ CAS Key Laboratory of Genomic and Precision Medicine, Beijing Institute of Genomics, Chinese Academy of Sciences and China National Center for Bioinformation, Beijing, China; ^2^ College of Life Sciences, University of Chinese Academy of Sciences, Beijing, China; ^3^ School of Future Technology, University of Chinese Academy of Sciences, Beijing, China; ^4^ Center for Precision Health, School of Biomedical Informatics, The University of Texas Health Science Center at Houston, Houston, TX, United States

**Keywords:** amyotrophic lateral sclerosis, causal variants, causal genes, cell type, transcriptome-wide association analysis, colocalization analysis, summary data-based Mendelian randomization analysis

## Abstract

Amyotrophic lateral sclerosis (ALS) is a fatal progressive multisystem disorder with limited therapeutic options. Although genome-wide association studies (GWASs) have revealed multiple ALS susceptibility loci, the exact identities of causal variants, genes, cell types, tissues, and their functional roles in the development of ALS remain largely unknown. Here, we reported a comprehensive post-GWAS analysis of the recent large ALS GWAS (*n* = 80,610), including functional mapping and annotation (FUMA), transcriptome-wide association study (TWAS), colocalization (COLOC), and summary data-based Mendelian randomization analyses (SMR) in extensive multi-omics datasets. Gene property analysis highlighted inhibitory neuron 6, oligodendrocytes, and GABAergic neurons (Gad1/Gad2) as functional cell types of ALS and confirmed cerebellum and cerebellar hemisphere as functional tissues of ALS. Functional annotation detected the presence of multiple deleterious variants at three loci (9p21.2, 12q13.3, and 12q14.2) and highlighted a list of SNPs that are potentially functional. TWAS, COLOC, and SMR identified 43 genes at 24 loci, including 23 novel genes and 10 novel loci, showing significant evidence of causality. Integrating multiple lines of evidence, we further proposed that rs2453555 at 9p21.2 and rs229243 at 14q12 functionally contribute to the development of ALS by regulating the expression of *C9orf72* in pituitary and *SCFD1* in skeletal muscle, respectively. Together, these results advance our understanding of the biological etiology of ALS, feed into new therapies, and provide a guide for subsequent functional experiments.

## Introduction

Amyotrophic lateral sclerosis (ALS, OMIM#105400) is a progressive neurological degenerative disorder without effective treatment affecting 1 in 400 individuals worldwide (van Rheenen et al., 2016). With the fast pace of global aging, ALS is anticipated to reach 380,000 cases globally by 2040 (Logroscino and Piccininni, 2019). The heritability of ALS has been estimated at around 0.61 (95% CI: 0.38–0.78) in a twin study (Al-Chalabi et al., 2010). Genome-wide association studies (GWASs, Supplementary Table S1) have identified more than 35 genetic loci associated with risk of ALS (van Es et al., 2009; van Rheenen et al., 2016; Nicolas et al., 2018; Project MinE ALS Sequencing Consortium, 2018; Zhou et al., 2018; Iacoangeli et al., 2020; Xiao et al., 2020), with 16 loci being identified in at least two GWASs, representing the most robust genetic associations. Identifying causal genetic variants, causal genes, and cell or tissue site of action remains a challenging task as over 90% of the ALS-associated variants fall in noncoding regions with largely unknown functions (Farh et al., 2015). Recently, three post-GWASs (Du et al., 2018; Xiao et al., 2020; Park et al., 2021) proposed lists of genes with high probabilities of causality, providing a better understanding of the genetic basis of the pathogenesis of ALS. However, the exact identities of causal variants, genes, cell types, and tissues remain largely unknown, leaving alone the complex causal relationships between them.

Here, we report a comprehensive functional characterization of the susceptibility loci identified in the large ALS GWAS (*n* = 80,610) (Nicolas et al., 2018) using functional mapping and annotation (FUMA). Furthermore, we systematically applied transcriptome-wide association analysis (TWAS), colocalization (COLOC), and summary data-based Mendelian randomization analysis (SMR) to prioritize putative causal genes using 18 publicly available eQTL datasets.

## Materials and Methods

ALS GWAS summary data reported by Nicolas et al. (2018) were downloaded from ALS Variant Server (http://als.umassmed.edu). This dataset incorporated several previous cohorts of ALS for meta-analysis, mainly including previous 20,806 cases and 59,804 controls of European ancestry. We harmonized GWAS summary statistics to the 1,000 reference genomes. After removing SNPs with low MAF (<0.01), our post-GWAS analysis included a total of 9,657,890 SNPs.

### Gene-Based Analysis, Tissue Specificity, and Cell Type

We performed a gene-level enrichment analysis using the MAGMA in FUMA platform v1.3.6a (Watanabe et al., 2017). MAGMA gene-property analysis was performed to assess relationships between tissue-specific or cell-specific gene expression profiles and ALS–gene associations (Watanabe et al., 2019). In cell-specific analysis, a 3-step workflow was performed with scRNA-seq datasets, 1) cell type analysis for each dataset was carried out, and significant cell types were retained for the next step; 2) step-wise conditional analyses to identify independent cell types within each dataset; and 3) cross-datasets conditional analysis was performed to examine the extent of similar association signals from significant cell types retained from the second step. For scRNA-seq datasets, PsychENCODE and DropViz were available for analysis.

### Functional Mapping and Annotation

The bioinformatic functional analysis was performed to investigate the functional relevance for ALS through FUMA (Watanabe et al., 2017), using the following toolsets or datasets, including ANNOVAR, CADD, RegulomeDB, eQTLGen, GTEx v8, and Hi-C data. Independent significant SNPs, lead SNPs, and genomic risk loci were defined to use the default parameters in FUMA. A CADD score with a threshold of 12.37 for a variant was considered deleterious (Kircher et al., 2014). The lower a RegulomeDB score, the more likely it is to be a regulatory element. Mapping SNPs to genes used 3 options including positional mapping, eQTL mapping, and chromatin interaction mapping. We restricted to eQTLGen, whole blood, and 13 brain tissues from GTEx v8 in eQTL mapping. Only significant SNP-gene pairs with FDR correction (*p* < 0.05) were identified to map genes. Chromatin interaction mapping was restricted to PsychENCODE, enhancer-promoter (EP) correlations from FANTOM5, adult cortex, and fetal cortex. The significance threshold of interaction was set at FDR < 1 × 10^−6^.

### Transcriptome-Wide Association Study

We applied S-PrediXcan (Barbeira et al., 2018) to test the association between the predicted expression levels of 13 brain tissues, skeletal muscle, pituitary, and whole blood from GTEx v8 and ALS, respectively. S-MultiXcan (Barbeira et al., 2019) integrated multiple tissue panels to improve the power to detect ALS-associated genes. Prediction models trained on GTEx v8 were obtained on the PredictDB website (http://predictdb.org). Genes with association FDR < 0.05 were considered to be significantly associated with ALS. A circus plot for multiple TWASs was created by the R package “circlize” (Gu et al., 2014).

We compared the identified loci with the published TWASs and GWASs (Supplementary Table S1, *p* threshold < 5 × 10^−8^), which was visualized with the R package “VennDiagram” (Chen and Boutros, 2011). However, the latest TWAS (Megat et al., 2021) was unavailable in the medRxiv and not included in the comparison.

### Colocalization Analysis

We applied COLOC (Giambartolomei et al., 2014) to assess the probability of the same variant being responsible for ALS risk and gene expression. Default conservative priors, p_1_ (1 × 10^−4^), p_2_ (1 × 10^−4^), and p_12_ (1 × 10^−5^) for a causal SNP in either ALS or gene expression and a shared causal SNP. We assessed the posterior probability of colocalization between ALS and Brain-eMeta (Ng et al., 2017), 13 brain tissues, skeletal muscle, pituitary, and whole blood from GTEx v8, eQTLGen, respectively. For three eQTL datasets, cis-association analyses were defined for SNPs within 1 MB of the transcription start site or the center of the probe (Qi et al., 2018; Võsa et al., 2021; Aguet et al., 2020). A detailed summary of eQTL data is in Supplementary Table S2. The regions in colocalization assessment had at least one SNP with a *p* value < 1 × 10^−6^ from GWAS that had a *p* value < 1 × 10^−4^ in the eQTL dataset. We applied suggested combination cutoffs (PP4 ≥ 0.75, PP3 + PP4 ≥ 0.9, and PP4/PP3 ≥ 3) as powerful evidence supporting a causal role for the gene to be a mediator of ALS risk. The regional association plots were generated by R package “LocusCompareR” (Liu et al., 2019).

### Summary Data-Based Mendelian Randomization Analysis and HEIDI Test

SMR (Zhu et al., 2016) was performed to evaluate the association between an exposure and an outcome in Mendelian randomization analysis principles by removing nongenetic confounding using a variant as an instrument variable. We used the same tissue as in the COOC analysis. A Benjamini–Hochberg method correction (FDR < 0.05) was used in each SMR analysis. HEIDI was applied to distinguish pleiotropy from linkage, and a threshold (HEIDI > 0.01) was considered to have little evidence of heterogeneity. We used SMR with the following parameters: *p-value* threshold *p* < 1 × 10^−6^ to select the top eQTL for the SMR test, a threshold for the difference in SNP allele frequency between datasets was set to 1, and other parameters were default options.

## Results

### Study Design and Analysis Workflow

**FIGURE 1:**
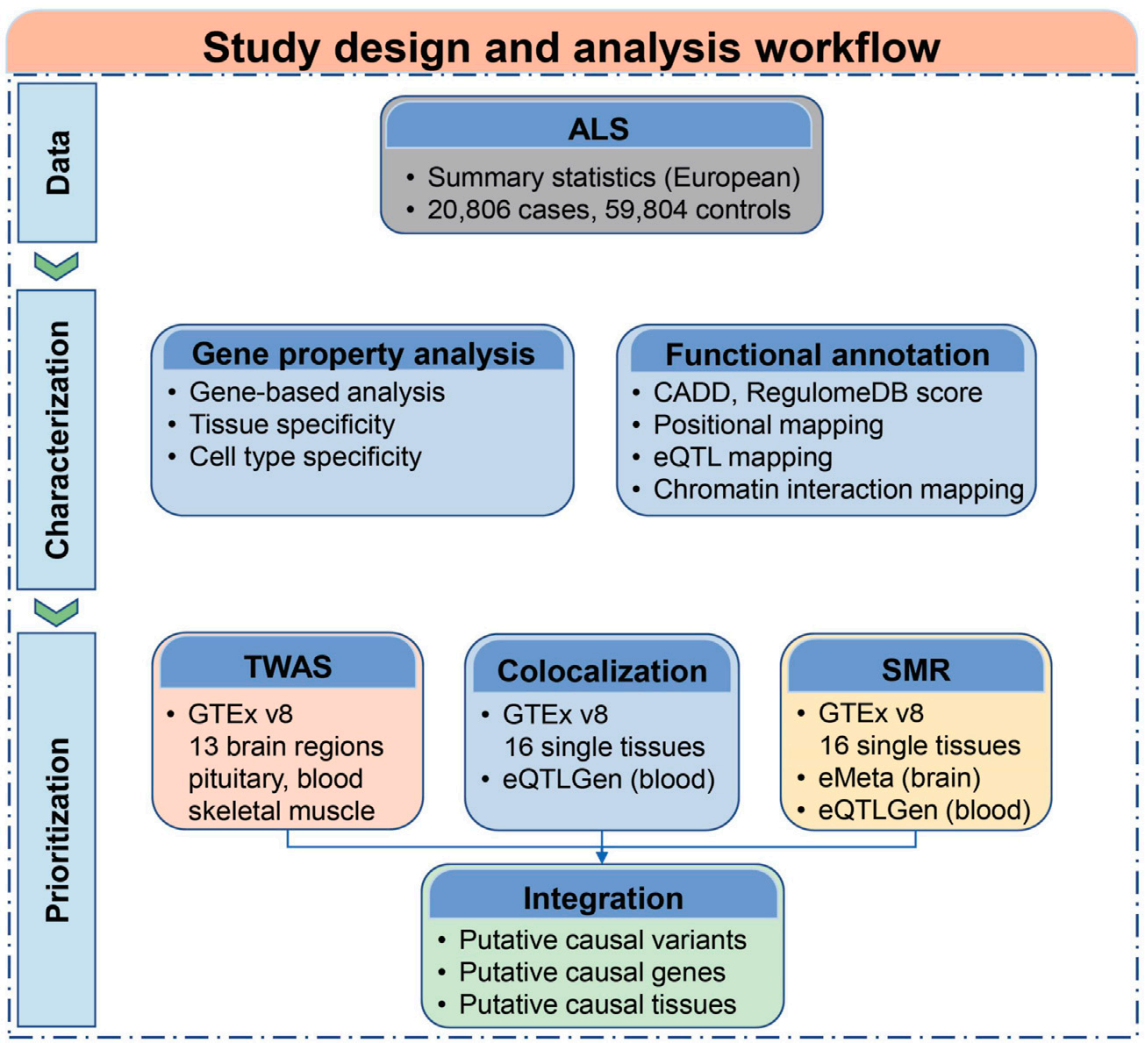
Schematic overview of the study design. Data, data collection and preprocessing. Characterization, characterizing ALS risk loci, including gene property analysis and functional annotation. Prioritization, putative causal genes prioritization and summarizing the evidence.

**FIGURE 2:**
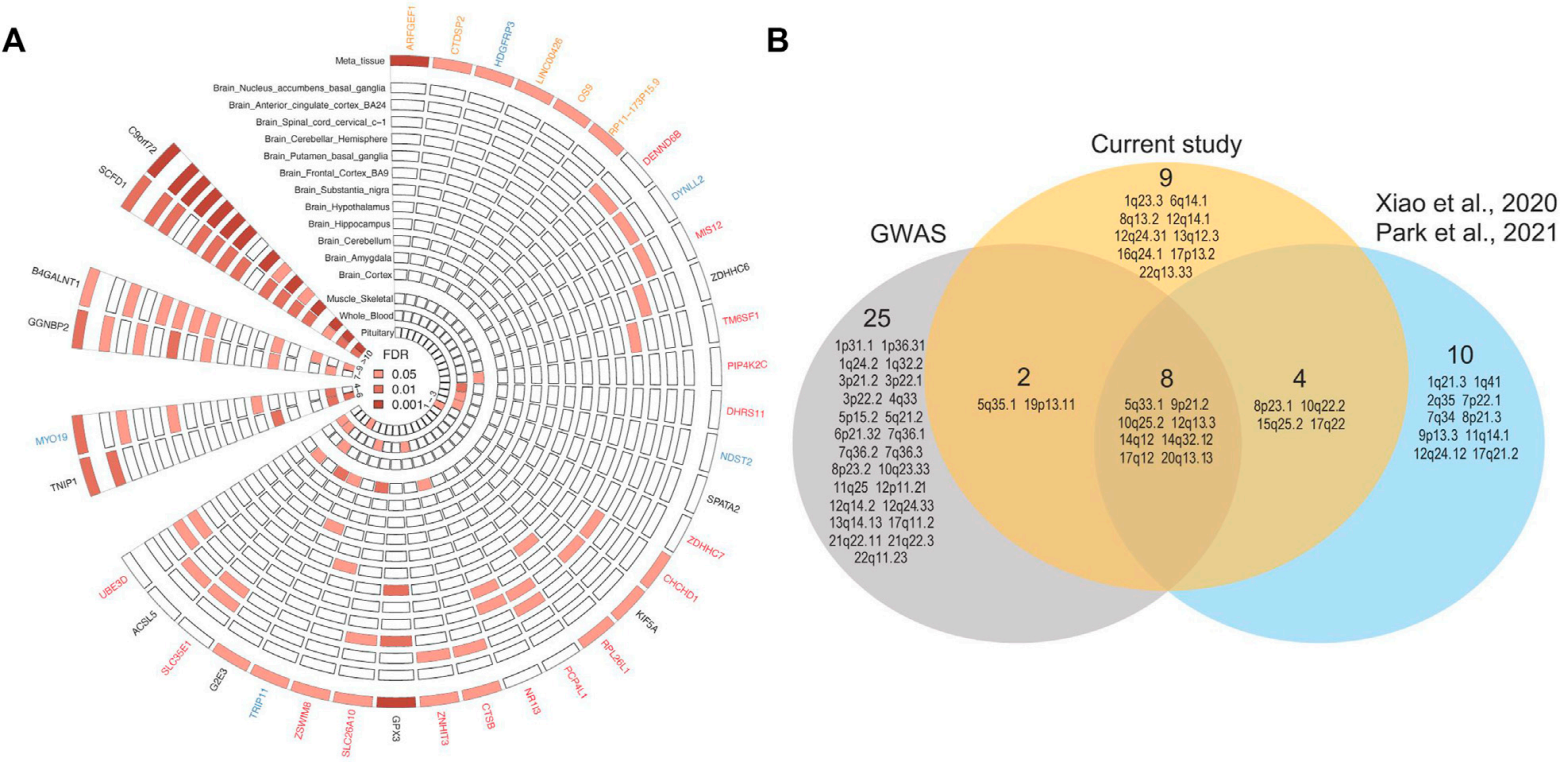
Identification of genes associated with ALS by TWAS analysis and comparison with other studies. **(A)** Genes associated with ALS were identified by S-PredictXcan and S-MetaXcan across tissues (FDR < 0.05). Meta_tissue represented the joint effect of gene expression from different tissues using S-MetaXcan. The circles represented different tissues from outside to inside, where the brain tissues were clustered together, while each sector expressed a different gene. The strength of color of each cell indicated the significance of the association of genes (sectors) with ALS in different tissues (circles). Among these genes, red and orange, respectively, indicate genes newly discovered from S-PrediXcan and S-MultiXcan, blue indicates the five replicated TWAS-discovered genes recently, and black indicates genes previously reported by GWAS. Brain tissues are grouped, and grouped genes with similar TWAS patterns are shown (the number of significant signals labeled in the innermost ring). **(B)** Venn diagram displayed loci identified in different studies. The gray circle represents published GWASs, the blue circle represents two recent TWASs from Xiao et al. (2020) and Park et al. (2021), and the orange circle represents our current TWAS. The number represents the number of loci in each region.

A schematic overview of the study design is illustrated in Figure 1. We conducted a survey of 26 GWASs (Dunckley et al., 2007; Schymick et al., 2007; Van Es et al., 2007; Cronin et al., 2008; Van Es et al., 2008; Landers et al., 2009; van Es et al., 2009; Laaksovirta et al., 2010; Shatunov et al., 2010; Kwee et al., 2012; The ALSGEN Consortium, 2013; Deng et al., 2013; Diekstra et al., 2014; Fogh et al., 2014; Xie et al., 2014; McLaughlin et al., 2015; Chen et al., 2016; Fogh et al., 2016; van Rheenen et al., 2016; Benyamin et al., 2017; Nicolas et al., 2018; Dekker et al., 2019; Wei et al., 2019; Iacoangeli et al., 2020; Nakamura et al., 2020) of ALS in the NHGRI-EBI GWAS Catalog (till September 2021) and additionally included one most recent GWAS (van Rheenen et al., 2021) and 3 recent post-GWASs (Du et al., 2018; Xiao et al., 2020; Park et al., 2021) to summarize the current knowledge on the candidate genes of ALS (Supplementary Table S1; Figure 2B). We then based our post-GWAS analyses on ALS GWAS from Nicolas et al. (2018), which represents the largest-ever GWAS data for ALS, totaling 20,806 cases and 59,804 controls of European ancestry. We first characterized a large set of possible tissues and cell types potentially functionally involved in the development of ALS by conducting a gene property analysis using FUMA. We next annotated the potential functions of a set of candidate genes using CADD score, RegulomeDB score, relative physical positioning with ALS-associated SNPs, evidence of eQTL, and chromatin interactions. We further prioritized the causality for a list of candidate genes and tissues by applying TWAS, COLOC, and SMR to 18 eQTL datasets from GTEx v8, eMeta, and eQTLGen. We finally integrated the findings from different methods and provided a list of variants and genes in corresponding tissues with high probabilities of causality.

### Gene Property Analysis Highlights a List of Tissues and Cell Types

The FUMA has implemented the MAGMA tissue-specificity analysis using 54 tissues from GTEx. We identified 13 brain regions and pituitary with nominally significant association with ALS–gene associations (*p* < 0.05), and two regions showed significance after Bonferroni correction: cerebellum (*p* = 2.7 × 10^−5^) and cerebellar hemisphere (*p* = 1.3 × 10^−4^) (Supplementary Figure S2). We thus focused on the 13 brain regions and pituitary in our subsequent analyses and additionally included skeletal muscle, which has been implied in ALS progression (Loeffler et al., 2016; Badu-Mensah et al., 2020), and whole blood, which was also implied in previous studies (Park et al., 2021).

MAGMA cell-type specificity analyses in a total of 196 cell types were performed in 8 different mouse and human scRNA-seq datasets. In the PsychENCODE, we identified both excitatory and inhibitory neurons as significantly (FDR < 0.05) associated with ALS (Supplementary Figure S3A; Supplementary Table S3), meaning that in these cells gene expression profiles were significantly associated with ALS-gene associations. Within-dataset step-wise conditional analyses identified inhibitory neuron 6 showing independent association (Supplementary Figure S3B; Supplementary Tables S4, S5), further highlighting the likelihood of inhibitory neuron 6 being the basic functional unit of ALS. In seven mouse datasets, within-dataset conditional analyses additionally identified oligodendrocytes and gamma-aminobutyric acidergic (GABAergic) neurons (Gad1/Gad2) as significantly associated cell types (Supplementary Figure S3A; Supplementary Tables S3–S5). Cross-dataset conditional analysis highlighted that inhibitory neuron 6, oligodendrocytes, and GABAergic neurons (Gad1/Gad2) are likely driven by distinct genetic signals, while various oligodendrocytes from different datasets are likely driven by similar genetic signals (Supplementary Figure S3C; Supplementary Table S6).

In addition, MAGMA identified seven genes showing significant association with ALS, and all have been identified in previous GWASs (Supplementary Figure S1).

### Functional Annotation of ALS-Associated SNPs and Genes

We annotated the functionality of 233 ALS-associated candidate SNPs from the 6 independent genome-wide significant loci (5q33.1 rs10463311, 9p21.2 rs3849943, 12q13.3-14.1 rs142321490, 12q14.2 rs74654358, 19p13.11 rs12973192, and 21q22.3 rs75087725) (Kircher et al., 2014). Of 233 candidate SNPs, CADD identified a total of 17 SNPs at 3 loci (9p21.2, 12q13.3, and 12q14.2) with high scores (>12.37), suggesting a strong deleterious effect of these variants (Supplementary Table S7). At 9p21.2, 14 SNPs had high scores tightly surrounding *C9orf72*, with the highest score observed for rs3736319 (18.5), 83 bp upstream of *MOB3B*, and 7.6 kbp downstream of *C9orf72*, and the second-highest score observed for rs10967965 (17.2), an intronic variant of *MOB3B*. At 12q14.1, two SNPs had high scores, including a UTR5 variant of *NAB2* (rs185306972) and a nonsynonymous variant of *KIF5A* (rs113247976). At 12q14.2, one synonymous variant of *TBK1* (rs41292019) had a high score. RegulomeDB (Boyle et al., 2012) further revealed three SNPs at 9p21.2 with strong evidence of regulation supported by eQTL and TF binding/DNase peak (evidence level 1f, Supplementary Table S7). All three were located in the intronic region of *MOB3B* and very close to *C9orf72* (<60 kbp), with one (rs10967965) also highlighted in the CADD analysis. These results provided evidence for the presence of deleterious variants with pathogenic effects and SNPs with regulatory effects in three ALS-associated loci and provided the list of candidate genes in these loci.

Positional mapping, eQTL mapping, and chromatin interaction mapping were mapped to 58 genes, among which 4 genes were mapped by all three mapping methods, including 5q33.1 *TNIP1*, 9p21.2 *C9orf72*, *MOB3B*, and *IFNK* (Supplementary Table S8). Interestingly, different from other loci, the 9p21.2 locus clearly contained a DNA loop (Fudenberg et al., 2016) in brain tissues (Supplementary Figure S4), which made parts of DNA closer together and allowed genes to be activated by regulatory elements known as enhancers. The two loci (12q14.1 and 12q14.2) contained more signals for both eQTL and chromatin interactions compared with the other four loci (Supplementary Figure S5). These results provided direct evidence for a list of SNPs and genes that are potentially functionally involved in the development of ALS.

### Multi-Tissue TWAS Identified Novel Functional Candidate Genes

S-PrediXcan found a total of 31 genes at 19 distinct loci showing significant (FDR < 0.05) association with ALS risk in at least one tissue. Among the 19 loci, 5 (1q23.3, 6q14.1, 16q24.1, 17p13.2, and 22q13.33) are newly identified (Figure 2B), highlighting six genes (*NR1I3*, *PCP4L1*, *UBE3D*, *ZDHHC7*, *MIS12*, and *DENND6B*). In addition, 16 genes have not been previously suggested as functional candidates for ALS (Figure 2A; Supplementary Table S10). Among the 31 genes, the most significant finding was *C9orf72* (FDR = 5.03 × 10^−18^ in Brain_Nucleus_accumbens_basal_ganglia), which was at orders of magnitude more significant than any other gene in any tissue (minimum FDR = 0.001). *C9orf72*, representing the most well-established gene involved in the risk of ALS, was significant not only in 11 brain regions but also in the pituitary, skeletal muscle, and blood. The second most significant finding was the gene *SCFD1* at 14q12, which also showed a significant association with ALS risk in 10 brain regions, pituitary, skeletal muscle, and blood (min FDR = 0.001 in Brain_Cerebellar_Hemisphere). The 16 newly identified genes had similar significance levels (with FDR ranging between 0.001 and 0.05), and all were significant in up to three tissues. Among these 16 genes, 13 from 11 loci were significant in at least one brain tissue, and 3 from 3 different loci were significant only in nonbrain tissues, that is, skeletal muscle (12q13.3 *PIP4K2C*), blood (17q12 *DHRS11*), and pituitary (16q24.1 *ZDHHC7*).

S-MultiXcan found a total of 22 genes at 14 distinct loci, among which six genes at six distinct loci were not identified in S-PrediXcan (Figure 2A; Supplementary Table S11), among which, five loci were novel. The most significant was *ARFGEF1* (FDR = 6.4 × 10^−4^), which involves vesicular trafficking and has previously been suggested to play a role in pathogenesis in ALS (Saris et al., 2009). Overall, our S-PrediXcan and S-MultiXcan together identified 21 novel genes at 15 novel loci, complementing the lists of previously established functional candidate genes and ALS-associated loci.

We additionally conducted a TWAS using two different methods, that is, a unified test for molecular signatures (UTMOST) (Hu et al., 2019) and joint-tissue imputation (JTI) (Zhou et al., 2020). As the key gene C9orf72 was not imputed in the UTMOST model, we focused on JTI. JTI identified a total of 18 genes (14 loci) in 16 tissues from GTEx v8 that reached the FDR < 0.05 significance level in the corresponding tissue. Among these, 11 genes (5q33.1 *TNIP1*, 9p21.2 *C9orf72*, 10q25.2 *ACSL5*, 12q13.3 *B4GALNT1*, 12q13.3 *PIP4K2C*, 14q12 *SCFD1*, 14q32.12 *TRIP11*, 17q12 *DHRS11*, 17q12 *ZNHIT3*, 17q12 *GGNBP2*, and 17q22 *DYNLL2*) at eight distinct loci overlapped with S-PrediXcan and S-MultiXcan (Supplementary Table S12). For the remaining seven genes, two genes (*SLC9A8* and *SNAI1*) were located at the locus 20q13.13, whereas S-PrediXcan identified a different gene *SPATA2*. One gene, *PLOD2* at 3q24, though failed to be identified by PrediXcan, was reported by our subsequent SMR analysis. The remaining four genes (4p16.3 *NSD2*, 5q22.1 *CAMK4*, 11p13 *AL356215.1*, and 20q11.22 *PIGU*) at four different loci have not been reported before (Supplementary Table S12).

### Colocalization Highlights Genotype-Mediated Genes in Corresponding Tissues

**FIGURE 3:**
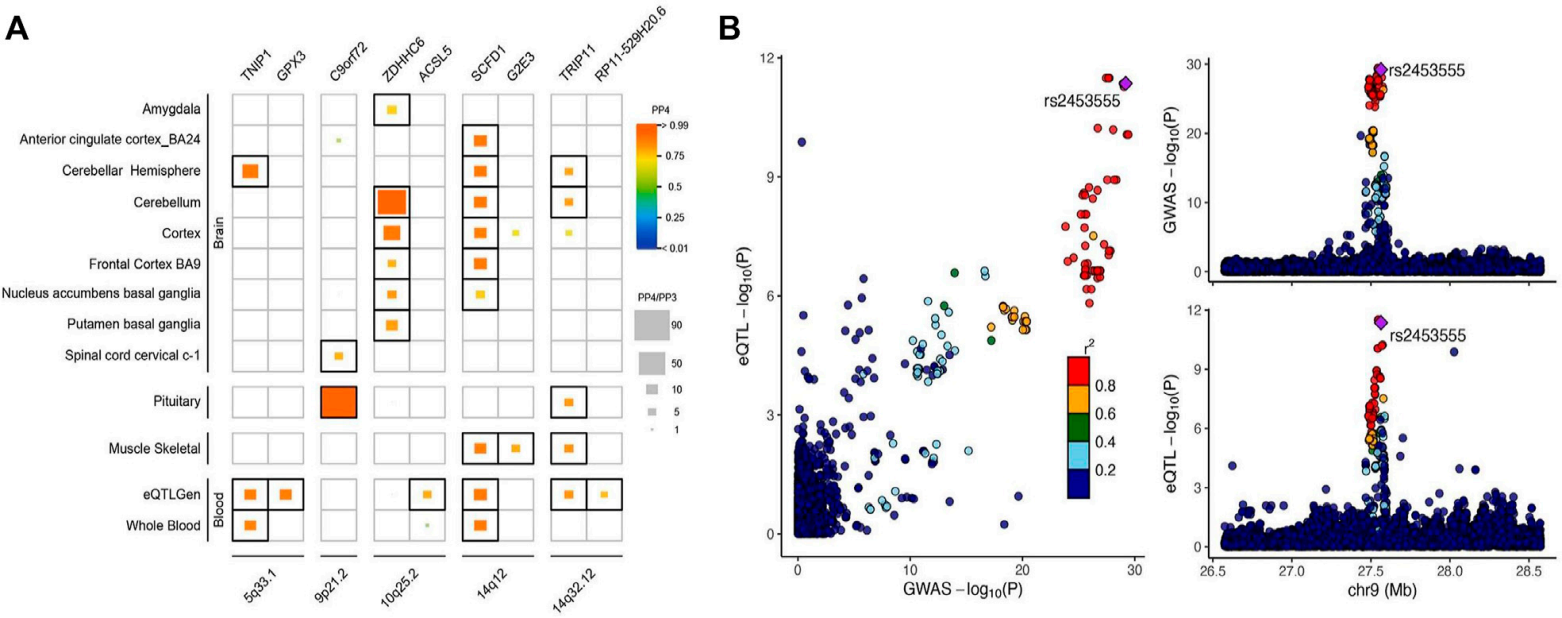
Colocalization of genetic ALS association and eQTL in different tissues. **(A)** Heatmap of significant colocalization (PP4 ≥ 0.75, PP3 + PP4 ≥ 0.9, and PP4/PP3 ≥ 3) in a total of 16 tissues analyzed. The horizontal axis represented genes under different cytobands, and the vertical axis represented different tissues, where brain regions and the blood tissues were lined up together, respectively. The cell color indicated the posterior probability of colocalization with orange indicating larger values, and the size of the inside squares was proportional to the PP4/PP3 ratio. **(B)** Illustration of the *C9orf72* locus in the pituitary (PP4 ≥ 0.99, PP3 + PP4 ≥ 0.99, and PP4/PP3 = 82.5). Each dot represented a genetic variant with the candidate causal variant, rs2453555, shown as a purple diamond. The color of other variants indicated their linkage disequilibrium (r2) based on the 1000 Genomes Project European reference panel with the purple diamond from blue to red. The left panel showed −log10 *p* values for SNP associations with ALS on the *x*-axes, and −log10 *p* values for associations with gene expression levels on the *y*-axes. The right panel illustrated genomic positions based on GRCh37 on the *x*-axes and −log10 *p* values of ALS GWAS (upper panel) and −log10 *p* values of gene expression at *C9orf72* in the pituitary gland (below panel) on the *y*-axes.

We conducted a series of eQTL colocalization analyses in 13 brain tissues, pituitary, skeletal muscle, and blood from GTEx v8 and eQTLGen. These analyses identified a total of 9 genes at 5 loci showing significant evidence (PP4 > 0.75, PP3 + PP4 > 0.9, and PP4/PP3 > 3, Figure 3A; Supplementary Table S13) of colocalization with eQTLs in at least one tissue. These included 5q33.1 (*TNIP1* and *GPX3*), 9p21.2 (*C9orf72*), 10q25.2 (*ZDHHC6* and *ACSL5*)*,* 14q12 (*SCFD1* and *G2E3*), and 14q32.12 (*TRIP11* and *RP11-529H20.6*).

The strongest signal according to the PP4/PP3 was identified for rs2453555 at 9p21.2 (PP4/PP3 = 82.5), which was highly significantly associated with ALS risk (GWAS *p* = 6.5 × 10^−30^), and at the same time served as a highly significant eQTL of *C9orf72* in the pituitary gland (eQTL *p* = 4.4 × 10^−12^), strongly suggesting a causal relationship (Figure 3B). This SNP was also a significant eQTL of *C9orf72* in spinal cord cervical at a much lower significance level (eQTL *p* = 8.1 × 10^−6^, PP4/PP3 = 5.2). This locus showed no evidence of colocalization with eQTLs in other tissues investigated. The SNP rs2453555 is in very high linkage disequilibrium (LD) with the most significant SNP (rs3849943, *p* = 3.8 × 10^−30^, *r* ^*2*^ = 0.98) in the GWAS of Nicolas et al. (2018). This result pinpoints rs2453555, which may regulate the expression of *C9orf72* in the pituitary, and consequently modifies the risk of ALS. A very recent study failed in finding colocalization signals for *C9orf72* (Mountjoy et al., 2021). Compared with their study, our study used the newest version of GTEx, which has an average 24% increased sample size.

The second strongest signal was observed for 10q25.2 *ZDHHC6* in the cerebellum (56.7) as well as in other five different brain tissues (5.1–19.9). The other gene (*ACSL5*) at this locus also showed significant colocalization (5.1) but at a much lower significance level than *ZDHHC6*, and the signal was observed only in blood. The third signal was 5q33.1 *TNIP1* with colocalization signals in the cerebellar hemisphere (18.0) and blood (9.7) but not in other tissues. The other gene at this locus (*GPX3*) was significant in only blood (10.0). The fourth signal was 14q12 *SCFD1* in six brain tissues, skeletal muscle, and blood at similar significance levels (PP4/PP3 ranging between 9.9 and 12 except in nucleus accumbens basal ganglia, where PP4/PP3 = 6.0). The other gene at this locus (*G2E3*) was detected only in skeletal muscle at a further decreased level of significance (5.5). The last signal was 14q32.12 *TRIP11* in the cerebellum, cerebellar hemisphere, pituitary, skeletal muscle, and blood with similar levels of significance (5.1–6.1). The other gene at this locus (*RP11-529H20.6*) showed a relatively weak colocalization signal in the blood (3.7). These results provided direct evidence of causality for a specific set of SNPs, genes, and corresponding tissues likely functionally involved in the development of ALS (Supplementary Table S13).

### SMR Illustrates the Causal Relationships Between SNPs, Gene Expression, and ALS Risk

We conducted a comprehensive SMR analysis for ALS based on the GWAS of Nicolas et al. (*n* = 80,610) in 9 brain tissues, pituitary, and skeletal muscle. We identified a total of 9 genes from 6 loci with significant evidence mediating the genetic associations observed in these loci (FDR < 0.05 and HEIDI > 0.01, Table 1). These included 3q24 *PLOD2*, 9p21.2 *C9orf72*, 10q22.2 *NDST2*, 14q12 *SCFD1*, 17q12 *GGNBP2*, *MYO19*, *DYNLL2*, *ZNHIT3*, and 22q13.33 *PLXNB2*. Among these 6 loci, 3q24 (*PLOD2* and rs149615181, skeletal muscle) and 22q13.33 (*PLXNB2* and rs62241220, blood) have not been previously reported. Interestingly, at 9p21.2, *C9orf72* was found highly significantly mediating the association between rs2453565 and the risk of ALS in the pituitary (FDR = 2 × 10^−5^), which was at orders of magnitude more significant than in other brain and nonbrain tissues. This SNP is in very high LD with rs2453555 (*r* ^*2*^ = 0.95) identified by our colocalization analysis. This result boosted the likelihood of a causal chain between rs2453555/rs2453565, expression of *C9orf72* in the pituitary, and the risk of ALS. In the recent study of van Rheenen et al. (2021), the HEIDI test rejected the hypothesis that expression of *C9orf72* could mediate the association between rs2453555 and ALS risk in blood. Our finding stresses the pituitary being the correct tissue where *C9orf72* plays a functional role in the development of ALS.

**TABLE 1:** Genes mediating the genetic associations with ALS in six loci from SMR and HEIDI analyses.

Locus	Gene	SNP	FDR	HEIDI	Tissue	Database
**3q24**	***PLOD2***	rs149615181	0.04	0.69	**Muscle_Skeletal**	GTEx v8
9p21.2	*C9orf72*	rs2453565	2.00 × 10^−5^	0.14	**Pituitary**	GTEx v8
		rs700795	0.02	0.19	**Brain_Spinal_cord_cervical_c-1**	GTEx v8
10q22.2	***NDST2***	rs11000785	0.05	0.07	Blood	eQTLGen
14q12	*SCFD1*	rs7144204	3.6 × 10^−3^	0.10	Blood	eQTLGen
		rs448175	0.01	0.35	Blood	GTEx v8
		rs229152	0.03	0.45	Brain_Cerebellum	GTEx v8
		rs229243	0.04	0.31	**Muscle_Skeletal**	GTEx v8
		rs2070339	0.03	0.22	Multiple brain regions	Brain-eMeta
17q12	*GGNBP2*	rs11650008	0.03	0.04	Blood	eQTLGen
	*MY O 19*	rs7222903	0.04	0.60	Blood	eQTLGen
	***DYNLL2***	rs2877858	0.04	0.16	Blood	eQTLGen
	***ZNHIT3***	rs4796224	0.05	0.50	Blood	eQTLGen
**22q13.33**	***PLXNB2***	rs62241220	0.02	0.77	Blood	eQTLGen

All genes with FDR < 0.05 and HEIDI > 0.01 are shown. Loci that have not been identified in previous GWASs or post-GWASs are indicated in bold. Genes that have not been reported in previous SMR studies are indicated in bold. Tissues that have not been reported in previous SMR studies are indicated in bold.

At 14q12, *SCFD1* in blood (FDR = 3.6 × 10^−3^ in eQTLGen), cerebellum (FDR = 0.03), and skeletal muscle (FDR = 0.04) showed significant mediatory effects on genetic association with ALS. Notably, rs229243 was detected to increase the risk of ALS by modifying *SCFD1* expression in skeletal muscle. This SNP is also a significant eQTL of *SCFD1* in skeletal muscle as found in our colocalization analysis (PP4/PP3 = 9.9). A recent SMR analysis (Iacoangeli et al., 2021) found that rs229243 had a regulatory effect on ALS risk mediated by the expression of *SCFD1* in the blood and cerebellum*.* Our SMR and colocalization results thus further provided evidence for skeletal muscle as an additional tissue possibly of function.

At 17q12, expressions of four genes (*GGNBP2*, *MYO19*, *DYNLL2*, and *ZNHIT3*) in the blood significantly mediated the genetic association in this locus (Supplementary Table S14). Among these four genes, *GGNBP2* was the most significant (FDR = 0.03), consistent with a previous study (Benyamin et al., 2017).

### Integration of Evidence Pinpoints Causal Genes in Corresponding Tissues

Overall, our study identified a total of 43 genes at 24 loci showing significant evidence of causality (Supplementary Table S15). Among these 43 genes, 23 genes at 17 loci have not been functionally linked with ALS in previous studies. Among the 24 loci, 10 loci (nine from TWAS, one from SMR) have not been associated with ALS risk in previous studies.

A total of eight genes at six loci were significant in at least two out of three analyses. These included 5q33.1 *TNIP1*, 9p21.2 *C9orf72*, 10q25.2 *ACSL5*, 10q22.2 *NDST2*, 14q12 *SCFD1*, 17q12 *MYO19*, *GGNBP2*, and *ZNHIT3*. All these six loci have been previously associated with ALS risk, and all eight genes have been previously suggested as the functional candidates. Integrating the results from three different analyses conducted in various tissues, our study further revealed their most likely corresponding functional tissues (Table 2).

**TABLE 2:** Integration of TWAS, COLOC, and SMR results.

Locus	Gene	Tissue	TWAS	COLOC (PP4/PP3)	SMR (HEIDI)	Overall evidence
5q33.1	*TNIP1*	Blood	2.2 × 10^−3^	0.91 (9.70)	3.6 × 10^−3^ (6.7 × 10^−4^)	∗∗
9p21.2	*C9orf72*	Brain_Spinal_cord_cervical_c-1	7.00 × 10^−13^	0.81 (5.10)	1.5 × 10^−2^ (0.19)	∗∗∗
	*C9orf72*	Pituitary	5.00 × 10^−14^	0.99 (82.50)	2.00 × 10^−5^ (0.14)	∗∗∗
10q25.2	*ACSL5*	Blood	2.7 × 10^−2^	0.82 (5.10)	—	∗∗
10q22.2	*NDST2*	Blood	3.3 × 10^−2^	—	4.9 × 10^−2^ (0.07)	∗∗
14q12	*SCFD1*	Brain_Cerebellum	1.7 × 10^−3^	0.92 (11.81)	3.1 × 10^−2^ (0.45)	∗∗∗
	*SCFD1*	Muscle_Skeletal	3.7 × 10^−2^	0.91 (9.85)	3.8 × 10^−2^ (0.31)	∗∗∗
	*SCFD1*	Blood	1.5 × 10^−3^	0.92 (12.00)	1.1 × 10^−2^ (0.35)	∗∗∗
	*SCFD1*	Brain_Anterior_cingulate_cortex_BA24	3.3 × 10^−3^	0.92 (11.85)	—	∗∗
	*SCFD1*	Brain_Cerebellar_Hemisphere	1.1 × 10^−3^	0.92 (11.35)	—	∗∗
	*SCFD1*	Brain_Cortex	2.4 × 10^−3^	0.91 (10.75)	—	∗∗
	*SCFD1*	Brain_Frontal_Cortex_BA9	1.3 × 10^−3^	0.92 (11.80)	—	∗∗
	*SCFD1*	Brain_Nucleus_accumbens_basal_ganglia	2.7 × 10^−3^	0.77 (6.01)	—	∗∗
17q12	*MY O 19*	Blood	2.2 × 10^−3^	—	4.0 × 10^−2^ (0.6)	∗∗
	*GGNBP2*	Blood	1.3 × 10^−2^	—	2.5 × 10^−3^ (0.04)	∗∗
	*ZNHIT3*	Blood	3.5 × 10^−2^	—	4.9 × 10^−2^ (0.5)	∗∗

Blood refers to whole blood from GTEx v8 or eQTLGen depending on which is more significant. The TWAS column indicates the *p-value* (FDR). The COLOC column indicates PP4 and PP4/PP3. The SMR column indicates the *p-value* (FDR) of the SMR test and the HEIDI test. The number of asterisks in the overall evidence column represents the number of significant results from three different analyses.

The most significant finding was for 9p21.2 *C9orf72*, which was highly significant in all three analyses, and all three analyses pinpointed pituitary as the most likely functional tissue, with orders of magnitude more significant than in any other tissues. We thus propose that in the pituitary, the expression of *C9orf72*, regulated by rs2453555, is causally associated with ALS risk.

14q12 *SCFD1* in the cerebellum, skeletal muscle, and blood were significant in all three analyses, and multiple other brain tissues were supported by two analyses, emphasizing the multi-tissue effect of *SCFD1*.

The remaining four loci (5q33.1, 10q25.2, 10q22.2, and 17q12) were supported by two analyses, but all suggesting blood instead of brain tissues being the causal tissue. This finding is somehow surprising and requires experimental validations in future studies. For 17q12, three genes (*MYO19*, *GGNBP2*, and *ZNHIT3*) are functional candidates. A previous study (Park et al., 2021) suggested *MYO19* as the most likely functional candidate of this locus, while another (Benyamin et al., 2017) suggested *GGNBP2*. Our analysis suggested that *GGNBP2* is less competitive with the other two as it had a more significant HEIDI (*p* = 0.04).

## Discussion

This study represents the most comprehensive post-GWAS of ALS to date. Our gene property analysis highlighted inhibitory neuron 6, oligodendrocytes, and GABAergic neurons (Gad1/Gad2) as functional cell types of ALS and confirmed cerebellum and cerebellar hemisphere as functional tissues of ALS. Functional annotation analysis detected the presence of multiple deleterious variants at 3 loci (9p21.2, 12q14.1, and 12q14.2) and highlighted a list of SNPs that are potentially functional. TWAS, COLOC, and SMR identified 43 genes at 24 loci, including 23 novel genes and 10 novel loci, showing significant evidence of causality. Integrating multiple lines of evidence identified that rs2453555 at 9p21.2 and rs229243 at 14q12 functionally contribute to the development of ALS by regulating the expression of *C9orf72* in pituitary and *SCFD1* in skeletal muscle, respectively.

Our gene property analysis identified a functional relationship between a novel cell type and ALS risk, especially inhibitory neuron 6. This finding is an important supplement to previous findings suggesting microglia (Clarke and Patani, 2020), astrocytes (Do-Ha et al., 2018; Saez-Atienzar et al., 2021), and glutamatergic neurons Cronin et al. (2008) as the key cell types functionally involved in the development of ALS. Our finding on inhibitory neurons is highly consistent with a recent study, which showed that genetic variants associated with ALS were mostly located in genes expressed in neurons, particularly in inhibitory neurons, and ALS risk loci were significantly enriched in excitatory and inhibitory neurons using single-cell assay for transposase-accessible chromatin sequencing (scATAC-seq) profiles (Megat et al., 2021). It is known that inhibitory neurons release the neurotransmitter gamma-aminobutyric acid (GABA) to regulate the initiation of excitatory neurons, ensuring our brain functions smoothly and accident-free (Foerster et al., 2013; Ramirez-Jarquin and Tapia, 2018). A loss of inhibitory neuron influence is an important factor leading to ALS pathogenesis (Turner and Kiernan, 2012).

Integrating multiple lines of evidence, we propose that rs2453555 at 9p21.2 functionally contribute to the development of ALS by regulating the expression of *C9orf72* in the pituitary. This SNP is the top significant in both COLOC and SMR (rs2453555 and rs2453565 in high LD with top rs3849943) analysis, strongly suggesting a functional role. This SNP tagging a highly pathogenic repeat expansion (GGGGCC) is also in high LD with multiple pathogenic variants at 9p21.2, although the possibility of the presence of multiple causal variants at this locus cannot be excluded. The aggregation of dipeptide repeats proteins (DPRs) originating from the *C9orf72* repeat expansion could result in disorders of hormone secretion and regulation in the pituitary, followed by disruption of the hypothalamic-pituitary axis (Pellecchia et al., 2010; Dedeene et al., 2020). Our identification of pituitary was highly consistent in our TWAS, COLOC, and SMR analysis. The failure of identification of pituitary and rs2453555 in the recent two studies (Mountjoy et al., 2021; van Rheenen et al., 2021) is likely because only blood and cortex or a smaller eQTL dataset were used. In addition, the cervical spinal cord was also of interest to another tissue as a similar pattern of rs2453555. A recent neuroimaging study reported that significant cerebral white matter (WM) atrophy was detected at every cervical vertebral level of *C9orf72* hexanucleotide expansion carriers (Querin et al., 2019). In contrast, another study showed that the cervical spinal cord progressively occurs to thin in ALS patients with *C9orf72* repeat expansion (van der Burgh et al., 2019). This discrepancy could be due to many factors, such as sample size, imaging techniques, and statistical analysis methods.

Multiple lines of evidence support that rs229243 at 14q12 functionally contributes to the development of ALS by modifying the expression of *SCFD1* in the skeletal muscle, providing a possible functional tissue. *SCFD1* is involved in vesicular transport between the endoplasmic reticulum and the Golgi (Hou et al., 2017). Surprisingly, skeletal muscle was also found to be the relevant tissue for *SCFD1*. Although the weakening of skeletal muscle was thought to be the initial hallmark of ALS, whether ALS originates in peripheral tissues (dying-back phenomenon) (Dadon-Nachum et al., 2011), including skeletal muscle or motor neurons (dying-forward phenomenon) (Braak et al., 2013) has been fiercely debated. Skeletal muscle was not considered pivotal to the etiology and treatment of ALS until recent years (Loeffler et al., 2016). Current studies about skeletal muscle degeneration/regeneration process focused on mutant *SOD1* mouse mode to the pathology of ALS (Cheng et al., 2019; Badu-Mensah et al., 2020; Steyn et al., 2020), but to our knowledge, *SCFD1* has never been investigated in molecular biology experiments or genetic models. Our findings could contribute to the understanding of skeletal muscle pathology and may provide a new therapeutic target for ALS. In addition, we also found 23 novel functional candidate genes (Supplementary Table S15), among which 12 have supportive evidence from the literature.

However, our study is not without limitations. First, the sample size of ALS GWAS is relatively small compared with the latest GWAS (van Rheenen et al., 2021), a parallel to our study. The sample size in different tissues and types for eQTL tissues limited our ability to identify ALS-related genes and explore the search for causal genes in other pathologically relevant tissues. Second, LD structure (Giambartolomei et al., 2014) and gender differences (Aguet et al., 2020) in the sample may bias findings due to the unavailability of raw data. Third, with the constraints of currently available data, only cis-eQTL data were included in our analysis, which may miss the actual causal genes. Fourth are the tissue pleiotropy and cell-type heterogeneity. The disease rarely works in a single tissue. Some genes could exert a causal effect on disease in specific tissues or cell types different from our reference panel, which may introduce bias and incompleteness. Therefore, identification of the possible causal tissue or cell type of each gene may be a hot topic for future development, and single-cell RNA sequencing holds promise for more refined studies in the future. Finally, further biological function experiments are needed to confirm the biological role of these genes in the pathology of ALS.

In conclusion, we established causal relationships between genetic variants, candidate genes, functional cell types and tissues, and ALS risk. The prioritized genes and tissues serve as targets for future functional and drug discovery studies.

## Data Availability

The original contributions presented in the study are included in the article/Supplementary Material; further inquiries can be directed to the corresponding authors.

## Glossary

ALS: amyotrophic lateral sclerosis
LD: linkage disequilibrium
eQTL: expression quantitative trait loci
FTD: frontotemporal dementia
AD: Alzheimer’s disease
PD: Parkinson’s disease
FUMA: functional mapping and annotation
MAGMA: Multi-marker Analysis of GenoMic Annotation
CADD: Combined Annotation–Dependent Depletion
GWAS: genome-wide association study
TWAS: transcriptome-wide association analysis
MASHR: multivariate Adaptive Shrinkage in R
COLOC: colocalization
SMR: summary data-based Mendelian randomization analysis
HEIDI: heterogeneity in dependent instruments
FDR: false discovery rate
GTEx: Genotype-Tissue Expression
scATAC-seq: single-cell assay for transposase-accessible chromatin sequencing
scRNA-seq: single-cell RNA sequencing
OR: odds ratio
GABA_A_: gamma-aminobutyric acid type A
C9orf72: C9orf72-SMCR8 complex subunit
SCFD1: Sec1 family domain containing 1
TNIP1: TNFAIP3-interacting protein 1
G2E3: G2/M-phase-specific E3 ubiquitin protein ligase
B4GALNT1: beta-1,4-N-acetyl-galactosaminyltransferase 1
ZDHHC7: zinc finger DHHC-type palmitoyltransferase 7
MYO19: myosin XIX
DHRS11: dehydrogenase/reductase 11
GGNBP2: gametogenetin binding protein 2
SPATA2: spermatogenesis-associated 2
ZSWIM8: zinc finger SWIM-type containing 8
ACSL5: acyl-CoA synthetase long chain family member 5
UBE3D: ubiquitin protein ligase E3D
NDST2: N-deacetylase and N-sulfotransferase 2
ZNHIT3: zinc finger HIT-type containing 3
POLD2: DNA polymerase delta 2, accessory subunit
DYNLL2: dynein light chain LC8-Type 2
KIF3B: kinesin family member 3B
ARFGEF1: ADP ribosylation factor guanine nucleotide exchange factor 1
GPX3: glutathione peroxidase 3
RPL26L1: ribosomal protein L26-like one
CTDSP2: CTD small phosphatase 2
TRIP11: thyroid hormone receptor interactor 11
CHCHD1: coiled-coil-helix-coiled-coil-helix domain containing one
HDGFRP3: hepatoma-derived growth factor-related protein 3
CTSB: cathepsin B
SLC26A10: solute carrier family 26 member 10
LINC00426: long intergenic non-protein coding RNA 426
MOB3B: MOB kinase activator 3B
ZDHHC6: zinc finger DHHC-type palmitoyltransferase 6
ATXN3: ataxin 3
UNC13A: Unc-13 homolog A
C21orf2: chromosome 21 open reading frame 2
SOD1: superoxide dismutase one
