# Supplementary material for: Causal Inference of Genetic Variants and Genes in Amyotrophic Lateral Sclerosis
Source: Front Genet. 2022 Jun 22;13:917142. doi: 10.3389/fgene.2022.917142 (PMC9257137; doi:10.3389/fgene.2022.917142)
Supplement: Supplementary file 1 [file DataSheet1.docx]

Causal Inference of Genetic Variants and Genes in Amyotrophic Lateral Sclerosis

**Supplementary Material**

**Content:**

- **Functional information of the novel genes**
- **Supplementary Figure S1-S5**
- **Supplementary Table S1-S15**

**Functional information of the novel genes**

Our study identified a total of 23 novel functional candidate genes, among which 12 have supportive evidence from references. *DENND6B* (*DENN Domain Containing 6B*) belongs to the *DENND6* family acting as GDP/GTP exchange factor (GEF) to activate Rab-GTPases (Marat et al., 2011) and dysregulation of GEF activity or function is related to a variety of diseases, including neurodegeneration (e.g., ALS) and neurodevelopmental disorders (Droppelmann et al., 2014). *DYNLL2* (*Dynein Light Chain LC8-Type 2*), as one of several non-catalytic accessory components of the cytoplasmic dynein 1 complex, could be a prospective candidate gene associated with ALS from cytoplasmic dynein light chain LC8 phylogenetic tree (Shah, 2007). *PIP4K2C* (*Phosphatidylinositol-5-Phosphate 4-Kinase Type 2 Gamma*) has been reported to decrease mutant Huntington protein aggregation through increasing basal autophagy and may be a promising target to treat progressive neurodegenerative Huntington's disease (Al-Ramahi et al., 2017). *HDGFRP3* (*Hepatoma Derived Growth Factor-Related Protein 3*) enhanced DNA synthesis and may play a role in cell proliferation, and was inferred to be associated with another most common motoneuron disorder (spinal muscular atrophy) from MalaCards human disease database (Rappaport et al., 2017). *SLC26A10* (*Solute Carrier Family 26 Member 10*) was found to have a lower OR (0.1) value and suggestive association with ALS using truncating variants model in exome sequencing study (Farhan et al., 2019). Moreover, a regionally specific splicing QTL (sQTL) was identified in *SLC26A10* exon 12 in cerebellar hemisphere and cerebellum (Zhang et al., 2020). Accumulating evidence showed abnormalities in alternative splicing events have been associated with various neurodegenerative disorders, such as ALS (Da Cruz and Cleveland, 2011), AD (Buée. et al., 2000), which implies sQTL in *SLC26A10* is expected to be further verified in ALS. *MIS12* (*MIS12 Kinetochore Complex Component*) was identified to affect synaptic development in Drosophila, and developmental defects in embryonic neuromuscular junctions were found in *mis12* mutants (Zhao et al., 2019). *ZDHHC7* (*Zinc Finger DHHC-Type Palmitoyltransferase 7*) was shown to regulate partially neuronal development, plasticity and brain microstructural changes from the latest research (Kerkenberg et al., 2021). In ALS, the IRE1α-XBP1 and ATF6 pathways were strongly activated, which was confirmed by a large increase in the expression of target genes involved endoplasmic reticulum, including *OS9* (*OS9 Endoplasmic Reticulum Lectin*), while a different pattern existed in Alzheimer disease (AD) cases (Montibeller and de Belleroche, 2018). *SLC35E1* (*Solute Carrier Family 35 Member E1*) was found to be the concordant direction of methylation and gene expression changes through comparing between differentially methylated genes (DMGs) and differentially expressed genes (DEGs) from the spinal cord in ALS (Figueroa-Romero et al., 2012). Additionally, *PCP4L1* (*Purkinje Cell Protein 4 Like 1*) was thought to be a differentially acetylated protein in ALS and non-ALS spinal cords (Liu et al., 2013). These results indicate that the identification of additional biomarkers in the future could better our understanding of ALS pathology. A previous study showed that *CTSB* (*Cathepsin B*) was involved in motor neuron degeneration and decreased in human ALS compared with control (Kikuchi et al., 2003), but contradicting result was observed in mice in a recent study (Hunter et al., 2021). *ARFGEF1* (*ADP Ribosylation Factor Guanine Nucleotide Exchange Factor 1*) involved in vesicular trafficking has previously been suggested to play a role in pathogenesis in ALS based on Gene Ontology (Saris et al., 2009). The imbalance of gamma-aminobutyric acid (GABA) and related modulators has been regarded as an important factor in the pathogenesis of ALS (Diana et al., 2017). Further, a recent study suggested that *ARFGEF1* haploinsufficiency endosome composition and decreased neuronal surface postsynaptic GABA_A_R (gamma-aminobutyric acid type A) (Teoh et al., 2020).

**References**

Al-Ramahi, I., Giridharan, S.S.P., Chen, Y.C., Patnaik, S., Safren, N., Hasegawa, J., et al. (2017). Inhibition of PIP4Kgamma ameliorates the pathological effects of mutant huntingtin protein. *Elife* 6. doi: 10.7554/eLife.29123.

Buée., L., Bussière., T., Buée-Scherrer., V., Delacourte., A., and Hof., P.R. (2000). Tau protein isoforms, phosphorylation and role in neurodegenerative disorders. *Brain Res Brain Res Rev* 33(1)**,** 95-130. doi: 10.1016/s0165-0173(00)00019-9.

Da Cruz, S., and Cleveland, D.W. (2011). Understanding the role of TDP-43 and FUS/TLS in ALS and beyond. *Current opinion in neurobiology* 21(6)**,** 904-919. doi: 10.1016/j.conb.2011.05.029.

Diana, A., Pillai, R., Bongioanni, P., O'Keeffe, A.G., Miller, R.G., and Moore, D.H. (2017). Gamma aminobutyric acid (GABA) modulators for amyotrophic lateral sclerosis/motor neuron disease. *Cochrane Database Syst Rev* 1**,** CD006049. doi: 10.1002/14651858.CD006049.pub2.

Droppelmann, C.A., Campos-Melo, D., Volkening, K., and Strong, M.J. (2014). The emerging role of guanine nucleotide exchange factors in ALS and other neurodegenerative diseases. *Front Cell Neurosci* 8**,** 282. doi: 10.3389/fncel.2014.00282.

Farhan, S.M.K., Howrigan, D.P., Abbott, L.E., Klim, J.R., Topp, S.D., Byrnes, A.E., et al. (2019). Exome sequencing in amyotrophic lateral sclerosis implicates a novel gene, DNAJC7, encoding a heat-shock protein. *Nat Neurosci* 22(12)**,** 1966-1974. doi: 10.1038/s41593-019-0530-0.

Figueroa-Romero, C., Hur, J., Bender, D.E., Delaney, C.E., Cataldo, M.D., Smith, A.L., et al. (2012). Identification of epigenetically altered genes in sporadic amyotrophic lateral sclerosis. *PLoS One* 7(12)**,** e52672. doi: 10.1371/journal.pone.0052672.

Hunter, M., Spiller, K.J., Dominique, M.A., Xu, H., Hunter, F.W., Fang, T.C., et al. (2021). Microglial transcriptome analysis in the rNLS8 mouse model of TDP-43 proteinopathy reveals discrete expression profiles associated with neurodegenerative progression and recovery. *Acta Neuropathol Commun* 9(1)**,** 140. doi: 10.1186/s40478-021-01239-x.

Kerkenberg, N., Wachsmuth, L., Zhang, M., Schettler, C., Ponimaskin, E., Faber, C., et al. (2021). Brain microstructural changes in mice persist in adulthood and are modulated by the palmitoyl acyltransferase ZDHHC7. *Eur J Neurosci* 54(6)**,** 5951-5967. doi: 10.1111/ejn.15415.

Kikuchi, H., Yamada, T., Furuya, H., Doh-ura, K., Ohyagi, Y., Iwaki, T., et al. (2003). Involvement of cathepsin B in the motor neuron degeneration of amyotrophic lateral sclerosis. *Acta Neuropathol* 105(5)**,** 462-468. doi: 10.1007/s00401-002-0667-9.

Liu, D., Liu, C., Li, J., Azadzoi, K., Yang, Y., Fei, Z., et al. (2013). Proteomic analysis reveals differentially regulated protein acetylation in human amyotrophic lateral sclerosis spinal cord. *PLoS One* 8(12)**,** e80779. doi: 10.1371/journal.pone.0080779.

Marat, A.L., Dokainish, H., and McPherson, P.S. (2011). DENN domain proteins: regulators of Rab GTPases. *J Biol Chem* 286(16)**,** 13791-13800. doi: 10.1074/jbc.R110.217067.

Montibeller, L., and de Belleroche, J. (2018). Amyotrophic lateral sclerosis (ALS) and Alzheimer's disease (AD) are characterised by differential activation of ER stress pathways: focus on UPR target genes. *Cell Stress Chaperones* 23(5)**,** 897-912. doi: 10.1007/s12192-018-0897-y.

Rappaport, N., Twik, M., Plaschkes, I., Nudel, R., Iny Stein, T., Levitt, J., et al. (2017). MalaCards: an amalgamated human disease compendium with diverse clinical and genetic annotation and structured search. *Nucleic Acids Res* 45(D1)**,** D877-D887. doi: 10.1093/nar/gkw1012.

Saris, C.G., Horvath, S., van Vught, P.W., van Es, M.A., Blauw, H.M., Fuller, T.F., et al. (2009). Weighted gene co-expression network analysis of the peripheral blood from Amyotrophic Lateral Sclerosis patients. *BMC Genomics* 10**,** 405. doi: 10.1186/1471-2164-10-405.

Shah, P.R. (2007). Complex genetic approaches to neurodegenerative diseases. *University of London*.

Teoh, J., Subramanian, N., Pero, M.E., Bartolini, F., Amador, A., Kanber, A., et al. (2020). Arfgef1 haploinsufficiency in mice alters neuronal endosome composition and decreases membrane surface postsynaptic GABAA receptors. *Neurobiol Dis* 134**,** 104632. doi: 10.1016/j.nbd.2019.104632.

Zhang, Y., Yang, H.T., Kadash-Edmondson, K., Pan, Y., Pan, Z., Davidson, B.L., et al. (2020). Regional Variation of Splicing QTLs in Human Brain. *Am J Hum Genet* 107(2)**,** 196-210. doi: 10.1016/j.ajhg.2020.06.002.

Zhao, G., Oztan, A., Ye, Y., and Schwarz, T.L. (2019). Kinetochore Proteins Have a Post-Mitotic Function in Neurodevelopment. *Dev Cell* 48(6)**,** 873-882 e874. doi: 10.1016/j.devcel.2019.02.003.

**Supplementary Figures**


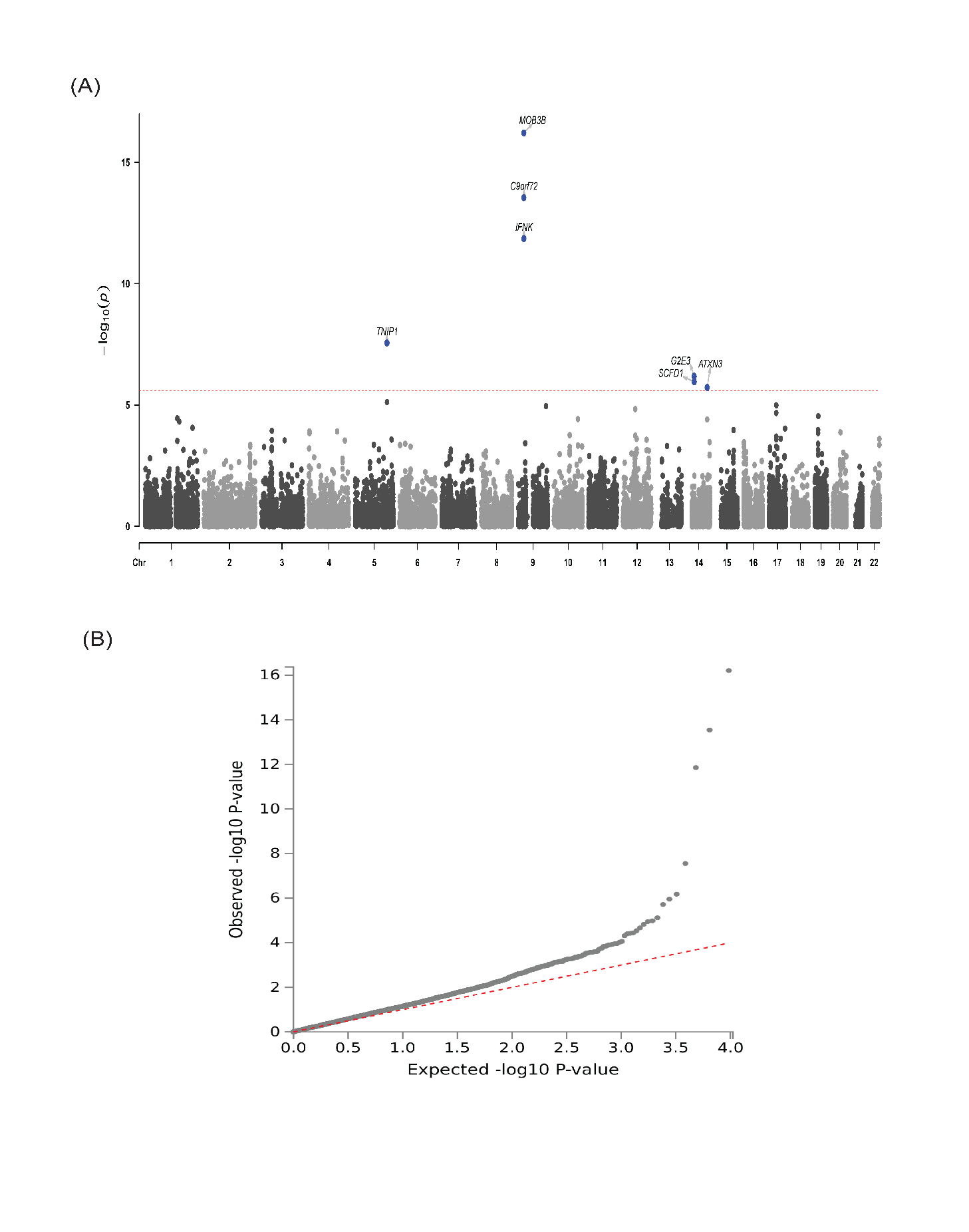


**Supplementary Figure S1.** Gene-based analysis for ALS GWAS computed by MAGMA **(A)** Manhattan plot of gene-based association tests. The dashed horizontal line indicated a genome-wide significance threshold of P<2.59 × 10^−6^ (0.05/19297). The significant locus was labeled in black. **(B)** The quantile-quantile (Q-Q) plot of the P values from gene-based tests for ALS. The expected P values (- log10 scale) were shown on the x-axis and the observed P values (- log10 scale) were shown on the y-axis.


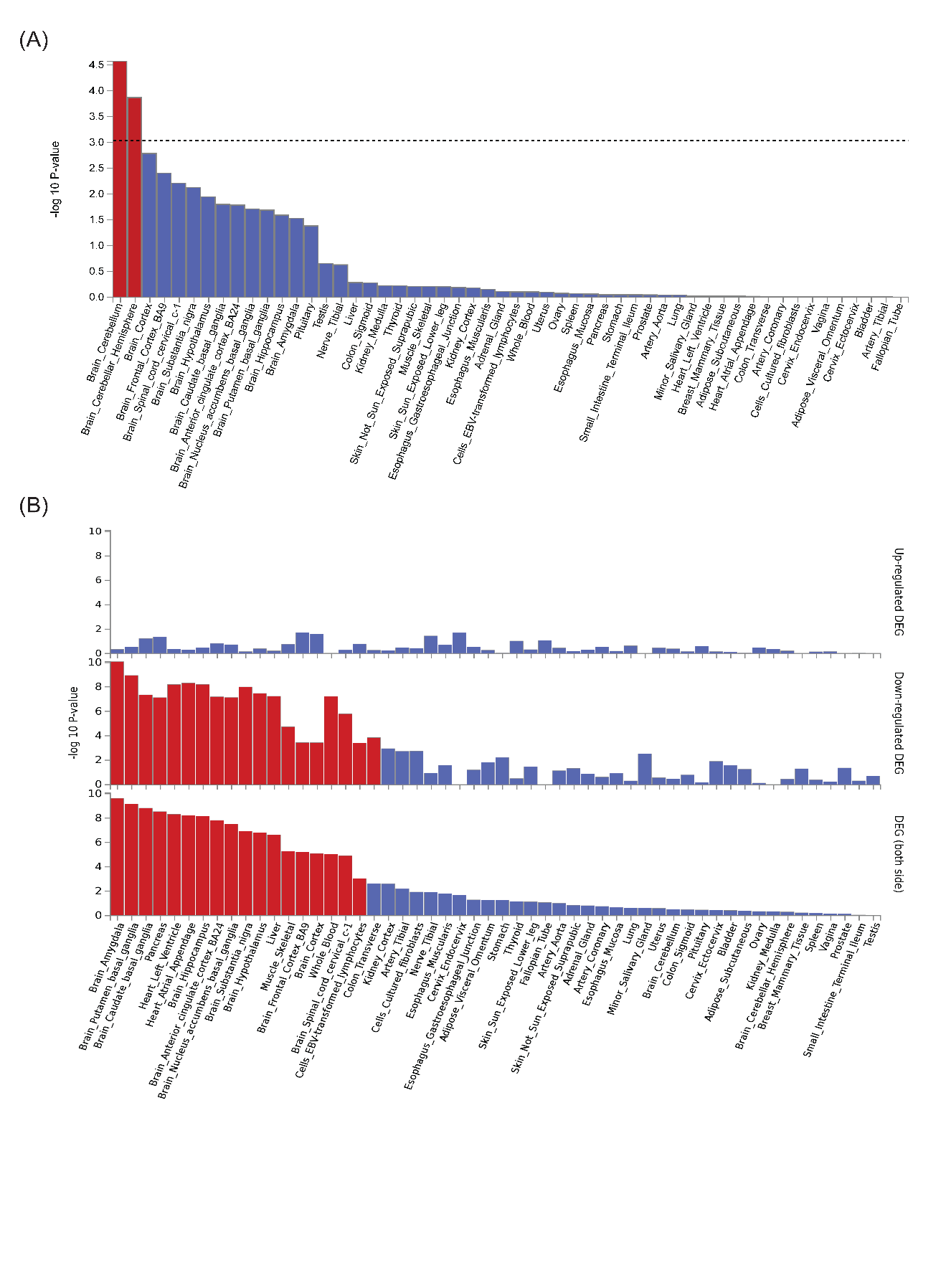


**Supplementary Figure S2.** Gene property analysis for tissue specificity of ALS using MAGMA. Gene expression datasets were from GTEx v8 54 tissues types. The y-axis shows −log10 P values for relationships between tissue specificity and genetic association of genes. The dashed horizontal line indicates Bonferroni correction threshold of P<9.26 × 10-4 (0.05/54).
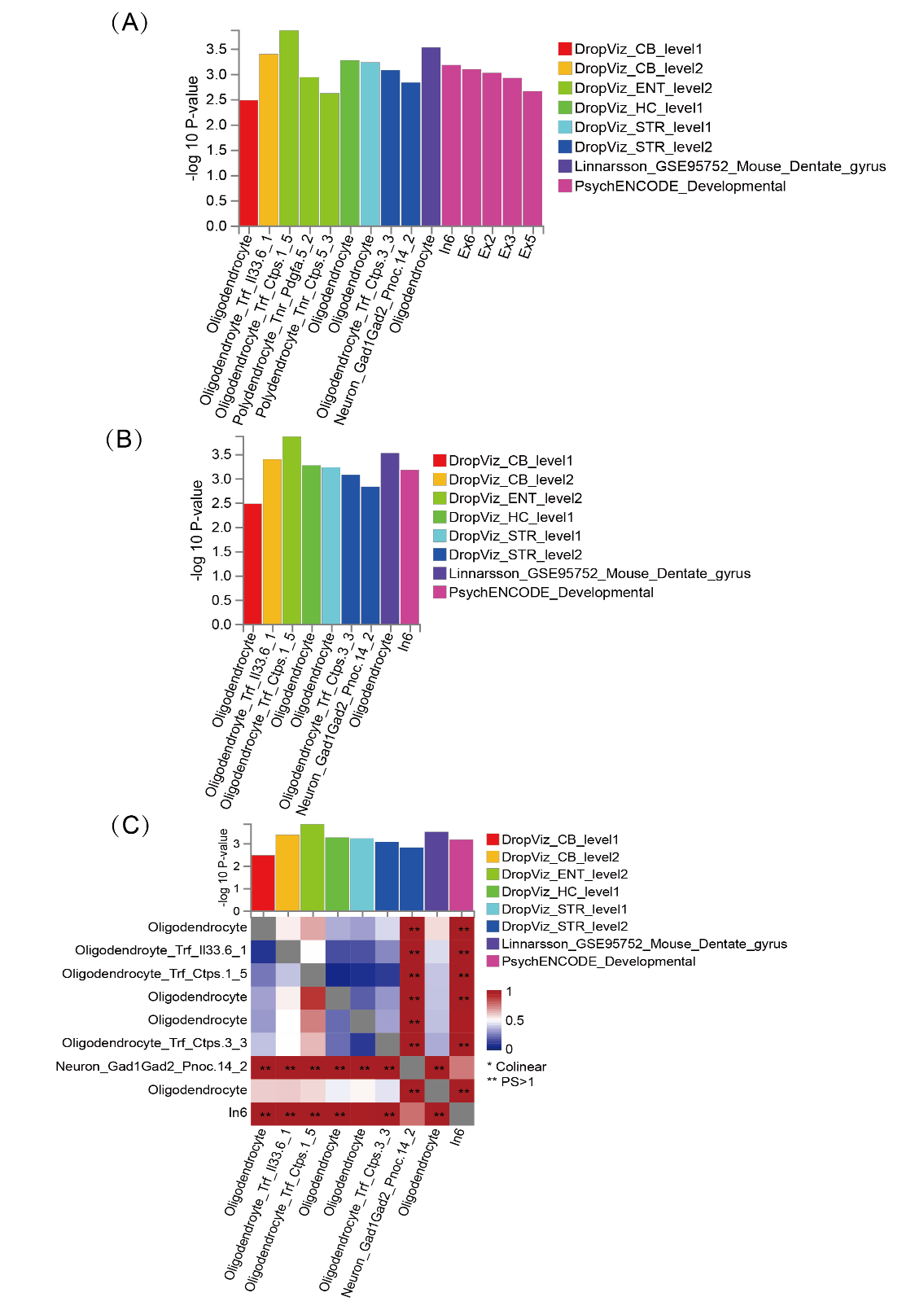


**Supplementary Figure S3.** Cell type specificity analyses with scRNA-seq. Gene expression data sets were from Human developmental (PsychENCODE), DropViz, and Linnarsson’s lab. The y-axis shows −log10 P values for relationships between cell type and genetic association of genes and red bars pass the FDR significance threshold corrected by FDR. This analysis contained three steps: (A) Cell type analysis for each dataset. FDR correction was performed separately for each dataset, respectively. Only the corrected cell type of each dataset was shown in the figure. Only significant cell types after FDR correction within the dataset indicated by different colors were present. (B) Independent cell type associations based on within-dataset conditional analyses. This analysis was performed for a dataset where more than one cell type reached significance. (C) Pair-wise cross-datasets conditional analyses. The top plot showed the same marginal P-value as the previous bar plot. Note that, the heat map below was not symmetrical. A cell in row i and column j was cross-dataset (CD) proportionally significant (PS), that was cell type j conditioning on cell type i. Each cell was colored by PS, where PS > 1 was indicated by double stars. A pair of cell types was represented by a star. The detailed analysis results can be found in Supplemental Table S3-S6. CB, Cerebellum; ENT, entopeduncular nucleus/subthalamic nucleus; HC, Hippocampus; STR, Striatum.


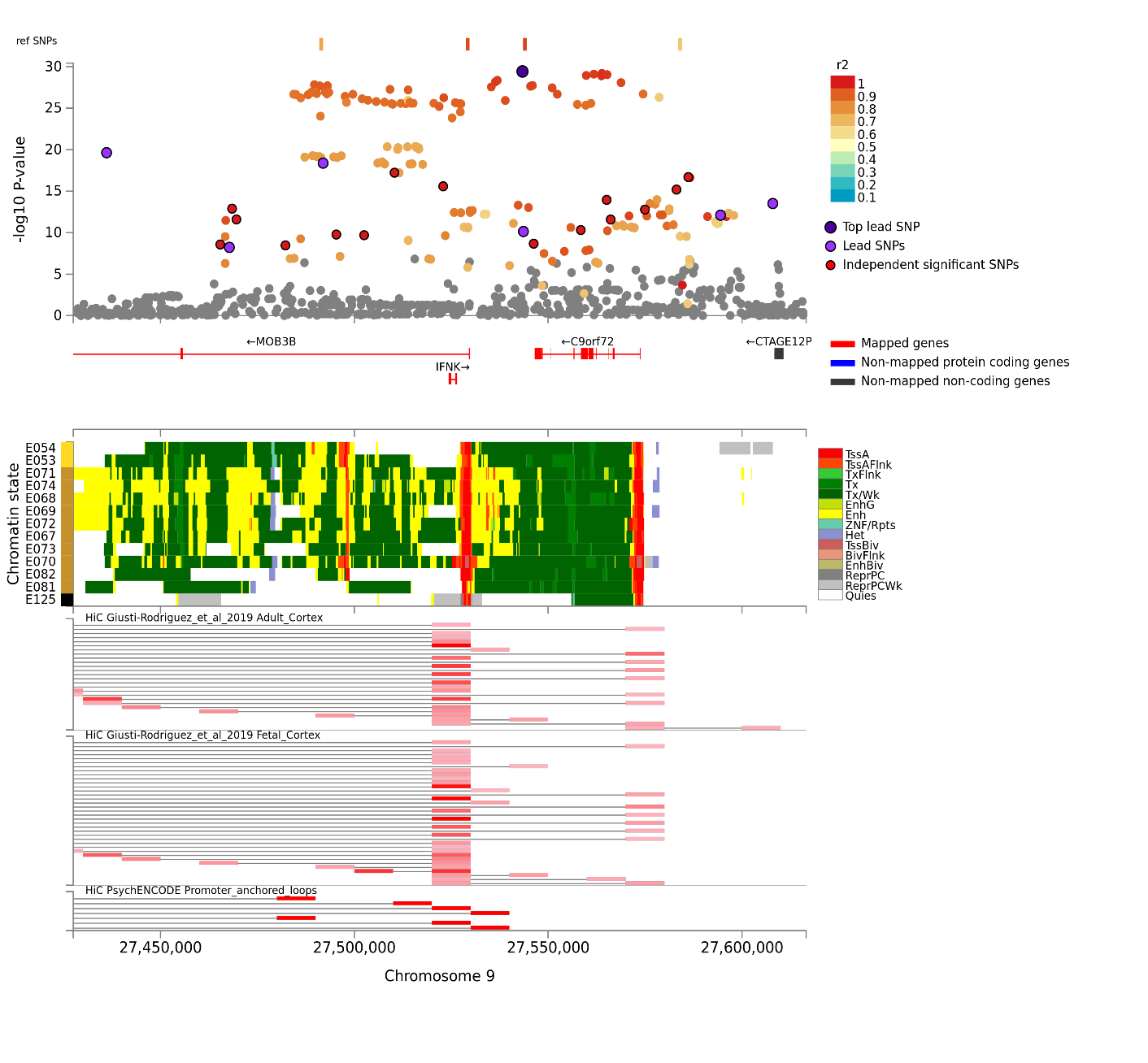


**Supplementary Figure S4.** Regional plot of *C9orf72* locus with annotation. Independent significant SNPs were defined as SNPs that have a genome-wide significance (P<5 × 10^-8^) and were independent of each other (LD r^2^<0.6). Lead SNPs were defined as independent significant SNPs and independent of each other (LD r^2^<0.1). 15 chromatin states predicted by ChromHMM were included in the middle plot. The below plot showed Chromatin interaction maps from Hi-C data, including adult cortex, fetal cortex (Giusti-Rodriguez et al.) and promoter anchored loops (PsychENCODE).


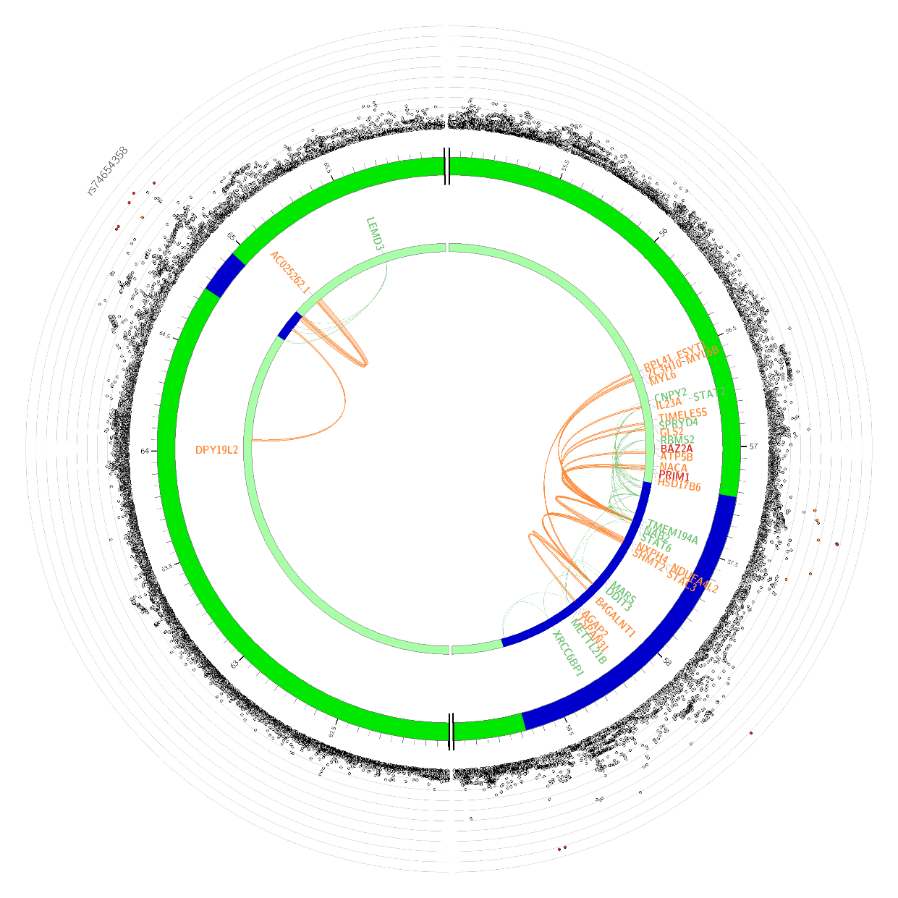


**Supplementary Figure S5.** Circos plot of chromatin interactions and eQTLs at risk locus in chromosome 12. The most significant SNP was marked in the outermost Manhattan plot of Circos plot, where only SNPs with p<0.05 were displayed. The color of the dots reflected the level of LD with the top SNP (red, r2>0.8; orange, r2>0.6; grey, r2<0.2). Genomic risk loci in the second or the third layer chromosome ring were highlighted in blue. Genes mapped by eQTL, mapped by Hi-C and both eQTL and Hi-C colored in orange, green and red, respectively.

**Supplemental Tables**

**Supplemental Table S1.** The review of previous genome-wide association studies (GWASs) and post GWASs for ALS. These results were summarized from GWAS Catalog (until September 01, 2021) and the latest references. N in Discovery/Replication N, the sample size of the case or control used in the discovery or replication if conducted. Genes, genes were mapped by associated SNPs at a significance threshold of P-value. The bolded genes were reported in at least one study. Genes identified by the gene-based analysis were shown in brackets. Asterisks indicated Post GWAS studies, including two recent TWAS studies and one SMR study. POP, population; EUR, European; EAS, East Asian.

**Supplemental Table S2**. The summary of gene-based analysis for ALS GWAS using MAGMA. The 1000 Genomes European phase 3 was linkage disequilibrium (LD) reference panel. The threshold for significance was corrected by Bonferroni correction (0.05/19297). CHR, Chromosomal location; START, Chromosomal start location of a gene; STOP, Chromosomal stop location of a gene; NPARAM, Number of relevant parameters used in the model; N, Sample size; ZSTAT, Z-scores derived from P-values; P, Gene level p values.

**Supplemental Table S3**. Cell type specificity analysis (step 1) of 8 datasets for ALS. The table contained all tested cell types in different datasets. FDR corrections were performed separately for each dataset. Dataset, Dataset name; Cell_type, Cell type name; NGENES, Number of genes used in the analysis; BETA, Effect size; BETA_STD, Standardized effect size; SE, Standard error; P, P-value; P.adj.pds, Adjusted P-value per dataset.

**Supplementary Table S4.** Conditional analysis of ALS (step 2) for each dataset. This table contained all possible pairs of cell types within each dataset that were significant after FDR correction. Dataset, Dataset name; Cell_type, Cell type name; MODEL, Index of the model. Cell types of the same MODEL index were conditioned on each other. BETA, Effect size; BETA_STD, Standardized effect size; SE, Standard error; P, P-value conditioned on the other cell type in the same MODEL index; Marginal.P, P-value without conditioning on the other cell type, which was the same as in the Table S3. PS, Proportional significance of the conditional P-value relative to the marginal P-value on a scale of log 10 (PS = -log10(P)/-log10(Marginal.P)).

**Supplementary Table S5.** Summary of Table S3 and Table S4 for ALS. This table contained all significant cell types after FDR correction within datasets. The first 8 columns were the same as in Table S3. Cond_state, The state of the conditional analysis for each dataset conditioned on the cell type in the "Cond_celltype" column. Cond_celltype, Conditioned cell type corresponding "Cond_state" column; In these two columns, the items were separated by semicolon in the respective order. For example, cell type ln6 had "joint; joint; joint; joint" in "Cond_state" column and "Ex2; Ex3; Ex5; Ex6" in "Cond_celltype" column, it meant ln 6 had state joint for cell type Ex2, Ex3, Ex5, Ex6, respectively. Step3, 1 if the cell type was retained to step 3 (cross-dataset conditional analysis), 0 otherwise.

**Supplementary Table S6.** Cross-datasets conditional analyses (step 3) for ALS. This table contained all possible pairs of cell types retained from Table S4. Dataset, Dataset name; Cell_type, Cell type name; MODEL, Index of the model; NGENES, Number of genes used in the analysis; BETA, Effect size; BETA_STD, Standardized effect size; SE, Standard error; P, Cross-datasets conditional P-value conditioned on the other cell type with the same MODEL index; CDM.BETA, Cross-datasets marginal effect sizes; CDM.BETA_STD, Cross-datasets marginal standardized effect size; CDM.SE, Cross-datasets marginal standard error; CDM.P, Cross-datasets P-value conditioned on the average of the dataset of other cell types with the same MODEL index; Marginal.P, the same as P-value in Table S3; PS, Proportional significance of the cross-dataset conditional P-value relative to the cross-dataset marginal P-value on a scale of log 10 (PS = -log10(P)/-log10(CDM.P)). When CDM.P was NA due to collinearity, PS=-log10(P)/-log10(Marginal.P); CDM.PS, Proportional significance when conditioning on the average expression from the other dataset (-log10(CDM.P)/-log10(Marginal.P)).

**Supplemental Table S7**. All candidate SNPs with annotations sorted by CADD scores. A variant with a CADD score greater than 12.37 was considered more deleterious. The RegulomeDB score ranging from 1a to 7 assessed the evidence for the regulatory potential and the lower a variant score, the more likely it to be a regulatory element. r2, The maximum r2 of the SNP with one of the independent significant SNPs; IndSigSNP, rsID of the independent significant SNP which has the maximum r2 with the SNP; nearestGene, The nearest Gene of the SNP based on ANNOVAR annotations; dist, SNP distance to the nearest gene; func, Functional consequence of the SNP on the gene obtained from ANNOVAR; minChrState, the minimum 15-core chromatin state across 127 tissue/cell type; commonChrState, The most common 15-core chromatin state across 127 tissue/cell types; posMapFilt, If the SNP was used for positional mapping 1, otherwise 0; exonic_func, Functional consequence of exonic SNPs (e.g., synonymous, stop gain).

**Supplemental Table S8.** The summary of mapped genes using three methods, including positional, eQTL and chromatin interaction mapping. ENSG, Ensembl gene; Symbol, Gene symbols; Chr, Chromosome; Start, Gene start; End, Gene end; Type; Gene type; HUGO, HUGO (HGNC) gene symbol; pLI, pLI score from ExAC database. The higher the score, the less tolerant the gene is to loss-of-function mutations. ncRVIS, non-coding residual variation intolerance score. The higher the score, the more tolerant the gene is to non-coding variants. posMapSNPs, Number of SNPs mapped to the gene based on positional mapping; eqtlMapSNPs, Number of SNPs mapped to the gene based on eQTL mapping; eqtlMapminP, The minimum eQTL P-value of mapped SNPs; eqtlMapts: Tissue types of mapped eQTL SNPs; ciMap, "Yes" if the gene was mapped by chromatin interaction mapping. ciMapts Tissue/cell types of mapped chromatin interactions. minGwasP, The minimum P-value of mapped SNPs. IndSigSNPs, rsID of all independent significant SNPs of mapped SNPs.

**Supplemental Table S9.** Significant eQTLs for SNPs associated with ALS (eQTL mapping). The table contained unique SNP-gene-tissue pairs so that an SNP could occur multiple times. db, Data source of eQTLs; p, P-value of eQTLs; Signed_stats, Signed statistics; RiskIncAllele, Risk increasing allele obtained from the input GWAS summary statistics; alignedDirection, The direction of effect to gene expression after aligning risk increasing allele of GWAS and tested allele of eQTLs. eqtlMapFilt, If the eQTL was used for eQTL mapping 1, otherwise 0.

**Supplemental Table S10**. The significant ALS-associated genes identified by S-PrediXcan across tissues. The eQTL datasets from 16 GTEx v8 tissues were used for this analysis. Significant genes were retained after multiple testing by FDR correction (P<0.05) in each tissue. cytoband, the position of the gene in the chromosome; Zscore, S-PrediXcan's association result for the gene; pvalue, P values of S-PrediXcan association. best_gwas_p, the highest p-value from GWAS SNPs used in this model; largest_weight, the largest (absolute value) weight in this model; p_fdr, P values for FDR multiple testing correction; Tissue, the tissue was used for the current analysis.

**Supplemental Table S11**. The significant ALS-associated genes identified by S-MetaXcan. 16 tissues from GTEx v8 were integrated for analysis. In each tissue, significant genes were kept after multiple testing by FDR correction (p<0.05). cytoband, the position of the gene in the chromosome; pvalue, significance p-value of S-MultiXcan association; n, number of tissues available for this gene; n_indep, number of independent components of variation kept among the tissues' predictions; p_i_best, best p-value of single-tissue S-PrediXcan association; t_i_best, name of best single-tissue S-PrediXcan association; p_i_worst, worst p-value of single-tissue S-PrediXcan association; t_i_worst, name of worst single-tissue S-PrediXcan association; eigen_max, eigenvalue (variance explained) of the top independent component in the SVD decomposition of predicted expression correlation; eigen_min, eigenvalue (variance explained) of the last independent component in the SVD decomposition of predicted expression correlation; eigen_min_kept, eigenvalue (variance explained) of the smallest independent component in the SVD decomposition of predicted expression correlation; z_min, minimum z-score among single-tissue S-Predican associations; z_max, maximum z-score among single-tissue S-Predican associations; z_mean, mean z-score among single-tissue S-Predican associations; z_sd, standard deviation of the mean z-score among single-tissue S-Predican associations; tmi, trace of T * T', where Tis correlation of predicted expression levels for different tissues multiplied by its SVD pseudo-inverse. It was an estimate for the number of independent components of variation in predicted expression across tissues (typically close to n_indep); status, If there was an error in the computation, it was stated here; p_fdr, P values for FDR multiple testing correction.

**Supplemental Table S12**. The significant ALS-associated genes identified by JTI. The eQTL datasets from 16 GTEx v8 tissues were used for this analysis. Significant genes were retained after multiple testing by FDR correction (P<0.05) in each tissue.

**Supplemental Table S13**. The summary of colocalization analysis between gene expression and ALS across tissues (sorted by PP4 descending). For this analysis, the eQTL datasets from 16 GTEx v8 tissues and eQTLGen, were used. ENSG, Ensembl gene; Gene, gene symbol; cytoband, the position of the gene in the chromosome; nsnps, Number of SNPs tested; PP0, Posterior probability of no causal SNP in the test region; PP1, Posterior probability of only one causal SNP for ALS GWAS; PP2, Posterior probability of only one causal SNP for gene expression; PP3, Posterior probability of two distinct causal SNPs in the test region; PP4, Posterior probability of sharing a causal SNP within the test region; PP3+PP4, Sum of PP3 and PP4; PP4/PP3, Ratio of PP4 to PP3; coloc_SNP, shared a single causal variant; Tissue, The tissue was used for the current analysis.

**Supplemental Table S14**. The significant ALS genes were identified by SMR in different datasets. Chr, Chromosome; topSNP, top SNP in eQTL; topSNP_bp, top SNP position; A1, effect allele; A2, other allele; Freq, effect allele frequency from reference population; b_GWAS, GWAS effect size; se_GWAS, GWAS standard error, p_GWAS, GWAS P-value; p_eQTL, eQTL P-value; b_SMR, SMR effect size; se_SMR, SMR standard error; p_SMR, SMR P-value, p_HEIDI, HEIDI (Heterogeneity in dependent instruments) test P value, nsnp_HEIDI, number of SNPs used in HEIDI test; p_SMR_fdr, P values for FDR correction. Tissue, The tissue was used for the current analysis.

**Supplemental Table S15**. Genes identified by TWAS, COLOC and SMR in different tissues. The first 7 columns were the same as in Table 2. Reference, supportive evidence for novel genes from literature in candidate review. Note, whether the gene was a new novel or reported. 9p21.2 *MOB3B*, *IFNK* and 14q32.12 *ATXN3* were replicated in MAGMA analysis, not in this table.
